# Update on the Development of Toehold Switch-Based Approach for Molecular Diagnostic Tests of COVID-19

**DOI:** 10.1155/2022/7130061

**Published:** 2022-05-09

**Authors:** Almando Geraldi, Ni Nyoman Tri Puspaningsih, Fatiha Khairunnisa

**Affiliations:** ^1^University CoE-Research Center for Bio-Molecule Engineering, Universitas Airlangga, Surabaya 60115, Indonesia; ^2^Department of Biology, Faculty of Science and Technology, Universitas Airlangga, Surabaya 60115, Indonesia; ^3^Department of Chemistry, Faculty of Science and Technology, Universitas Airlangga, Surabaya 60115, Indonesia

## Abstract

A high volume of diagnostic tests is needed during the coronavirus disease 2019 (COVID-19) pandemic to obtain representative results. These results can help to design and implement effective policies to prevent the spread of severe acute respiratory syndrome coronavirus 2 (SARS-CoV-2). Diagnosis using current gold standard methods, i.e., real-time quantitative PCR (RT-qPCR), is challenging, especially in areas with limited trained personnel and health-related infrastructure. The toehold switch-based diagnostic system is a promising alternative method for detecting SARS-CoV-2 that has advantages such as inexpensive cost per testing, rapid, and highly sensitive and specific analysis. Moreover, the system can be applied to paper-based platforms, simplifying the distribution and utilization in low-resource settings. This review provides insight into the development of toehold switch-based diagnostic devices as the most recent methods for detecting SARS-CoV-2.

## 1. Introduction

Since its first identification in late 2019 in Wuhan, the People's Republic of China, the severe acute respiratory syndrome coronavirus 2 (SARS-CoV-2) has caused a global pandemic of coronavirus disease 2019 (COVID-19) [1, 2]. In April 2022, the Center for Systems Science and Engineering (CSSE) at Johns Hopkins University (JHU) announced in their COVID-19 dashboard that the pandemic has led to more than 490 million confirmed cases with more than 6 million deaths worldwide [3]. Besides increasing the burden on public healthcare systems, COVID-19 negatively impacts the global economic and social conditions [4]. Preventive methods such as social distancing, lockdowns, and travel restriction policies were considered effective in limiting the spread of SARS-CoV-2. Unfortunately, the consequences of these methods have weakened the global economy [5, 6].

In late 2020, the COVID-19 vaccination program was started [7–9], and by April 2022, there were more than 11 billion doses of vaccine administered, with more than 64% of the world population having received at least one dose of the vaccine [10]. Although the number of vaccinated people kept increasing, preventive efforts to control the spread of COVID-19 via diagnostic tests are still critical. Efficient diagnostic tests are crucial to prevent the spread of SARS-CoV-2 infection by identifying positive individuals to be quarantined and preventing unnecessary quarantine of negative individuals [11, 12].

The most commonly used diagnostic tests for SARS-CoV-2 detection are nucleic acid amplification tests (NAATs), including reverse transcriptase real-time quantitative PCR (RT-qPCR). The workflow of RT-qPCR comprises the specimen sampling from patients, RNA extraction, conversion of purified RNAs to DNAs using reverse transcriptase (RT), and amplification of the obtained virus-originated DNA fragments. These fragments can detect unique viral RNA sequences in nucleocapsid (N), envelope (E), spike (S), or RNA-dependent RNA polymerase (RdRp) genes using the fluorescence signals [13, 14]. The mentioned method is currently considered the “gold standard” in COVID-19 diagnostics due to its high sensitivity and specificity and its straightforward quantitative analysis [15, 16]. However, SARS-CoV-2 diagnostic using RT-qPCR requires substantial hours and relatively expensive instruments, chemicals, and consumables to generate results [17]. Furthermore, the process involved, such as sample collection from patients, DNA purification and amplification, and result interpretation, requires skilled technicians, which increases the costs of the diagnostics [11, 18]. Consequently, various alternative techniques to detect SARS-CoV-2 RNA in patient's samples are being developed to obtain a more cost-efficient and rapid method.

## 2. Synthetic Biology-Based Diagnostics

Synthetic biology combines molecular biology and engineering approach to create new biological functions [19]. Synthetic biology has enabled the construction of biological systems rationally and systematically [20]. Furthermore, as the synthetic biology field matured, most components and parts to build novel biological systems, including diagnostic systems, are standardized and cataloged [21, 22]. Those facts, coupled with the availability of various biological data and the advancement of *in silico* biological analysis, have allowed for more straightforward, rapid, and inexpensive development of diagnostic systems. [23, 24].

Among the emerging synthetic biology-inspired diagnostic platforms for COVID-19 are toehold switch- and clustered regularly interspaced short palindromic repeats/CRISPR-associated- (CRISPR/Cas-) based systems. Both systems detect the genetic materials from pathogens and report the detection via visual signal.

**Figure 1:**
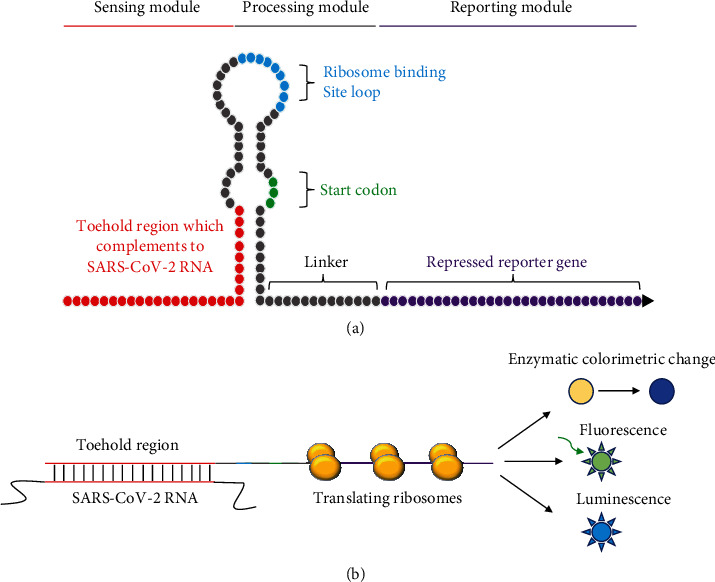
Toehold switch-based diagnostics for SARS-CoV-2. (a) General scheme of toehold switch riboregulator. In the absence of trigger RNA (SARS-CoV-2 RNA), RBS and start codon are hidden in the hairpin loop structure and inaccessible to the ribosome. (b) In the presence of SARS-CoV-2 RNA, the RBS and start codon are “released” to translate reporter genes, resulting in expression that acts as a signal detectable by naked eyes or specific instruments.

The toehold-based system uses an RNA switch containing sequences complementary to the target SARS-CoV-2 RNA (Figure 1(a)) [18, 25]. The binding of target SARS-CoV-2 RNA will activate the reporter gene's expression, resulting in a visually observed product. Meanwhile, the CRISPR/Cas-based systems involve CRISPR RNA (crRNA) that is specifically designed to bind the target pathogen DNA or RNA and activate the nonspecific cleavage activity of Cas nuclease (i.e., Cas12 and Cas13) to cleave quenched fluorescent DNA or RNA reporter [26–28]. The cleaved DNA or RNA reporter will emit a fluorescence signal which can be detected visually. Both systems can be applied to a paper-based and wearable platform that simplifies diagnosis, reduces analysis costs, and facilitates storage and deployment in areas lacking advanced infrastructures and medical experts [29].

Both systems have been developed to detect the presence of SARS-CoV-2 in patients' samples, and comprehensive reviews on the CRISPR/Cas-based system for COVID-19 diagnosis were reported [30–32]. This review focuses on discussing the application of toehold switches for COVID-19 diagnosis.

## 3. Toehold Switch-Based SARS-CoV-2 Diagnostic Systems

Toehold switches are riboregulators that control gene expression via base pairing with target RNA sequences. The main component of toehold switches is the RNA hairpin structure which contains the ribosome binding site (RBS) sequence and start codon (AUG) [33]. The upstream region of the hairpin comprises a single-stranded toehold sequence complemented with the target RNA (trigger RNA). The binding of trigger RNA to the toehold sequence will open the hairpin structure and release the RBS, initiating the downstream reporter gene's translation (Figure 1(b)) [34]. The advantage of using a toehold switch as an RNA biosensing device lies in the unpaired nature of the toehold sequence that can be designed to detect a wide range of trigger RNA, including full-length mRNA. Those features enable the utilization of riboregulators in various applications [35].

Pardee et al. [36] have successfully applied the toehold switch for diagnostic purposes. The portable diagnostic device consisted of a plasmid with the toehold switch and reporter gene encoding sequences, *in vitro* cell-free transcription, and translation systems that were immobilized onto paper or other porous materials [37]. The device successfully detected antibiotic resistance genes, Ebola virus, Zika virus, dengue virus, and various gut bacteria with high specificity and sensitivity [38, 39].

As demonstrated previously, the toehold switch-diagnostic devices were stable for long-term storage at room temperature, enabling the use of the device area with limited medical resources. Furthermore, such diagnostic devices can be designed based on the sequence of the genetic materials of the target pathogen, enabling a short design for the production cycle. Finally, the devices can cost as little as 0.04 USD per sensor using an in-house cell-free expression system than 4.00 USD (reagents only) for PCR-based tests [36, 38].

**Figure 2:**
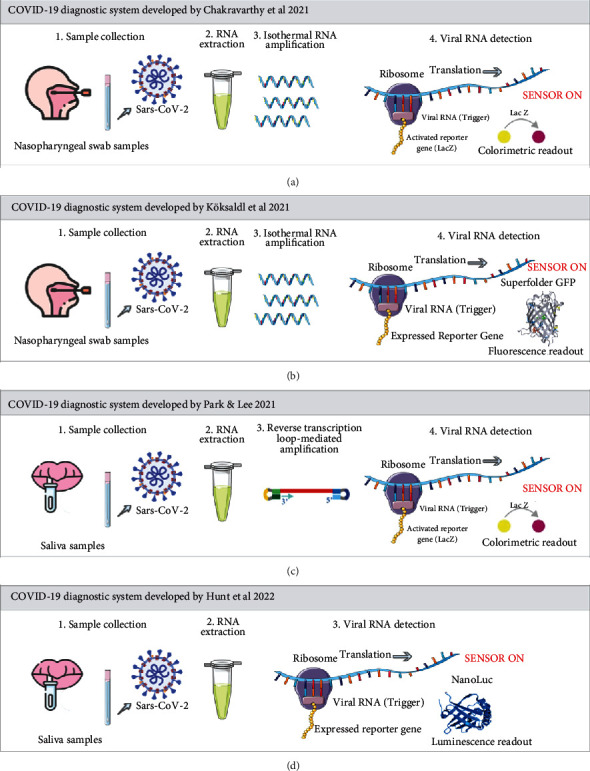
General scheme of the currently developed toehold switch-based diagnostics for COVID-19: (a) a system developed by Chakravarthy et al. [40] which utilized NASBA (nucleic acid sequence-based amplification) for amplifying trigger RNA from patient's nasopharyngeal swab sample and toehold switch-based biosensor with *lacZ* as a reporter gene, (b) a system developed by Köksaldl et al. [41] which utilized NASBA for amplifying trigger RNA from patient's nasopharyngeal swab sample and toehold switch-based biosensor with superfolder GFP as a reporter gene, (c) a system developed by Park et al. [42] which utilized reverse transcription loop-mediated amplification (RT-LAMP) for amplifying trigger RNA from patient's saliva sample and toehold switch-based biosensor with *lacZ* as a reporter gene, and (d) a trigger RNA amplification-free system developed by Hunt et al. [43] which detected SARS-CoV-2 RNA from patient's saliva sample using toehold switch-based biosensor with NanoLuc as a reporter gene.

The development of toehold switch-based COVID-19 diagnostic devices has been reported since 2021 (Table 1). Chakravarthy et al. [40] developed PHAsed NASBA-translation optical method (PHANTOM), a toehold switch-based biosensor coupled with isothermal NASBA (nucleic acid sequence-based amplification) to detect the SARS-CoV-2 genome (Figure 2(a)). In the PHANTOM system, RNA from SARS-CoV-2 in a patient's sample was extracted and then amplified isothermally using NASBA. A specifically designed toehold-based biosensor then detected the 36-nt amplification product (trigger RNA) in an *in vitro* transcription-translation (IVTT) assay. As a reporter gene, *lacZ* produces *β*-galactosidase to catalyze the colorimetric reaction of substrates such as ortho-nitrophenyl-*β*-galactoside (ONPG) or chlorophenol red-*β*-D-galactopyranoside (CPRG). The PHANTOM system can efficiently detect the presence of viral RNA in patient samples, which correlated well with the Ct value from the RT-qPCR test.

A similar system was developed by Köksaldl et al. [41] which coupled NASBA with a toehold switch-based biosensor to detect trigger sequences from the S gene and ORF1ab of SARS-CoV-2 that cost less than 1.00 USD per reaction. In contrast with PHANTOM, this diagnostic system uses a superfolder green fluorescent protein (sfGFP) as a reporter gene (Figure 2(b)). The system successfully detected the SARS-CoV-2 RNA from the nasopharyngeal swab sample in a relatively short period, i.e., 60 minutes via highly sensitive detectors of a microplate reader, or 2 hours through eye visibility with minimal requirement of 1800 viral RNA copies.

Park et al. [42] reported the development of a toehold switch-based COVID-19 diagnostic system with a relatively faster turnaround time than other similar systems. The diagnostic system coupled toehold switch-based biosensor with reverse transcription loop-mediated amplification (RT-LAMP) using *lacZ* as a reporter gene (Figure 2(c)). The diagnostic system detected SARS-CoV-2 RNA in the patients' saliva samples. The sensitivity of this system relies on its RT-LAMP strategy, which can amplify 120 copies of target SARS-CoV-2 RNA in 20 minutes. The short amplification time shortens the overall target RNA detection to only 70 minutes.

Finally, Hunt et al. [43] reported the toehold switch-based COVID-19 diagnostic device that did not require an amplification step. The system comprises lyophilized cell-free protein synthesis (CFPS) and toehold switch riboregulator that can detect capsid protein gene region in the SARS-CoV-2 genome with NanoLuc as a reporter gene (Figure 2(d)). The system detected the presence of SARS-CoV-2 RNA in saliva samples in just 7 minutes with an estimated cost of 0.50 USD. Similar to the system developed by Park et al., the components of this diagnostic device, i.e., toehold switch module and IVTT, were successfully immobilized in paper matrices which simplified the distribution and utilization of the COVID-19 diagnostic system in developed and developing areas.

## 4. Conclusion and Future Perspectives

The ongoing COVID-19 pandemic has emphasized the importance of diagnostic testing in outbreak control [44, 45]. Efforts to end the COVID-19 pandemic require the accurate utilization of diagnostic testing in high volumes and the rapid use of the results to help implement the appropriate therapy and policy, preventing further disease spread [46]. Conducting such high throughput diagnostic tests is challenging, especially in areas with limited health personnel and infrastructures. The World Health Organization has developed the ASSURED (affordable, sensitive, specific, user-friendly, rapid and robust, equipment-free, and deliverable to end-users) criteria as a benchmark for diagnostics tests in resource-limited settings [47].

As described in this review, the currently developed toehold switch-based COVID-19 diagnostic has met the ASSURED criteria with low-cost, rapid, and highly sensitive and specific analysis advantages. Toehold switch-based COVID-19 diagnostics also provide more logistical benefits and ease of use than currently used diagnosis methods such as RT-qPCR. Furthermore, the short design to the production cycle of the diagnostics offers a valuable edge to cope with the emergence of new variants of SARS-CoV-2 [48].

To date, there are no commercial toehold switch-based diagnostics for COVID-19. To produce a commercial diagnostic test kit, it must pass clinical testing and trials, including benchmarking against existing diagnostic tools to ensure the quality of the analysis results [27]. Additionally, the specificity and sensitivity of the toehold switch-based diagnostics for COVID-19 need to be evaluated in an in-field situation, where environmental conditions such as high temperatures, humidity, or dust might reduce the performance of the diagnostics [49].

## Figures and Tables

**Table 1:** Characteristics of currently developed toehold switch-based diagnostics for COVID-19.

Methods (references)	Amplification step	Viral RNA sources	Detection time	Observation results	Limit of detection	Estimated price per reaction
PHAsed NASBA-translation optical method (PHANTOM) [40]	Yes, isothermal NASBA (nucleic acid sequence-based amplification)	Nasopharyngeal swab samples	60-100 minutes	Naked eye, camera, and microplate reader	100 copies of viral RNA per sample	N/A
[41]	Yes, isothermal NASBA (nucleic acid sequence-based amplification)	Nasopharyngeal swab samples	60-120 minutes	Naked eye, camera, and microplate reader	1800 copies of viral RNA per sample	<1.00 USD
[42]	Yes, reverse transcription loop-mediated amplification (RT-LAMP)	Saliva	70 minutes	Naked eye, camera, and microplate reader	120 copies of viral RNA per sample	N/A
[43]	No	Saliva	Up to 7-12 minutes	Naked eye in the darkroom, camera	10 nM RNA per sample	<0.50 USD

## Data Availability

The data related to this article are available from the corresponding author upon request.
